# Resilience Informatics in Public Health: Qualitative Analysis of Conference Proceedings

**DOI:** 10.2196/63217

**Published:** 2025-01-16

**Authors:** Maiya G Block Ngaybe, Lidia Azurdia Sierra, Andrew McNair, Myla Gonzalez, Mona Arora, Kacey Ernst, Enrique Noriega-Atala, M Sriram Iyengar

**Affiliations:** 1 Health Promotion Sciences University of Arizona, Mel and Enid Zuckerman College of Public Health Tucson, AZ United States; 2 Department of Linguistics University of Arizona, College of Social & Behavioral Sciences Tucson, AZ United States; 3 Community, Environment & Policy Department University of Arizona, Mel and Enid Zuckerman College of Public Health Tucson, AZ United States; 4 Department Chair & Professor, Epidemiology and Biostatistics University of Arizona, Mel and Enid Zuckerman College of Public Health Tucson, AZ United States; 5 University of Arizona Tucson, AZ United States; 6 College of Medicine - Phoenix University of Arizona Phoenix, AZ United States

**Keywords:** resilience, public health, informatics, mobile phone, artificial intelligence, AI

## Abstract

**Background:**

In recent years, public health has confronted 2 formidable challenges: the devastating COVID-19 pandemic and the enduring threat of climate change. The convergence of these crises underscores the urgent need for resilient solutions. Resilience informatics (RI), an emerging discipline at the intersection of informatics and public health, leverages real-time data integration from health systems, environmental monitoring, and technological tools to develop adaptive responses to multifaceted crises. It offers promising avenues for mitigating and adapting to these challenges by proactively identifying vulnerabilities and fostering adaptive capacity in public health systems. Addressing critical questions regarding target audiences, privacy concerns, and scalability is paramount to fostering resilience in the face of evolving health threats.

**Objective:**

The University of Arizona held a workshop, titled Resilience Informatics in Public Health, in November 2023 to serve as a pivotal forum for advancing these discussions and catalyzing collaborative efforts within the field. This paper aims to present a qualitative thematic analysis of the findings from this workshop.

**Methods:**

A purposive sampling strategy was used to invite 40 experts by email from diverse fields, including public health, medicine, weather services, informatics, environmental science, and resilience, to participate in the workshop. The event featured presentations from key experts, followed by group discussions facilitated by experts. The attendees engaged in collaborative reflection and discussion on predetermined questions. Discussions were systematically recorded by University of Arizona students, and qualitative analysis was conducted. A detailed thematic analysis was performed using an inductive approach, supported by MAXQDA software to manage and organize data. Two independent researchers coded the transcripts; discrepancies in coding were resolved through consensus, ensuring a rigorous synthesis of the findings.

**Results:**

The workshop hosted 27 experts at the University of Arizona, 21 (78%) of whom were from public health–related fields. Of these 27 experts, 8 (30%) were from the field of resilience. In addition, participants from governmental agencies, American Indian groups, weather services, and a mobile health organization attended. Qualitative analysis identified major themes, including the potential of RI tools, threats to resilience (eg, health care access, infrastructure, and climate change), challenges with RI tools (eg, usability, funding, and real-time response), and standards for RI tools (eg, technological, logistical, and sociological). The attendees emphasized the importance of equitable access, community engagement, and iterative development in RI projects.

**Conclusions:**

The RI workshop emphasized the necessity for accessible, user-friendly tools bridging technical knowledge and community needs. The workshop’s conclusions provide a road map for future public health resilience, highlighting the need for scalable, culturally sensitive, community-driven interventions. Future directions include focused discussions to yield concrete outputs such as implementation guidelines and tool designs, reshaping public health strategies in the face of emerging threats.

## Introduction

### Background

Over the past few years, 2 major threats to public health have emerged. The recent COVID-19 pandemic caused an estimated 1 million deaths in the United States [1], and the long-term effects of COVID-19 are still being identified. Meanwhile, climate change is the long-term shift in predicted temperatures and weather behaviors for a given region [2]. While COVID-19 emerged within just a few months, making it an acute stressor, climate change’s effects have developed gradually, making it a chronic stressor [3]. In the long term, the public health effects of climate change could be even more serious as global warming, infectious disease, air pollution, and other consequences increase in severity [4-7]. The effects of climate change threaten the physical and mental health of the public via respiratory disease, infection, stress, malnutrition, heat exhaustion, and a variety of other negative consequences [4-7].

In the aftermath of the COVID-19 pandemic, public health is grappling with the growth of misinformation [8,9], persistent health inequities [10-12], a deepening mental health crisis [13,14], rising malnutrition rates [15,16], and a myriad of other critical challenges. There is now great pressure on the field of public health to prevent a future pandemic and control public response. In navigating a post–COVID-19 world, public health is now faced with the challenges of improving communication across disciplines [17,18], preventing the spread of misinformation and disinformation [19,20], responding quickly in the face of another public health emergency [21], addressing mental health issues [13,14], and tackling the nutritional crisis within the United States [15,16]. On a global scale, the health impacts of political tensions and climate change, particularly in relation to refugee displacement, add to these challenges [22,23].

To successfully cope with these inevitable threats to public health, people and communities need to develop resilience, defined as the ability of people, households, communities, countries, and systems to mitigate, adapt to, and recover from shocks and stresses in a manner that reduces chronic vulnerability and facilitates inclusive growth [24]. It is recognized that resilience is a necessary precursor to achieving the United Nations’ Sustainable Development Goals, including “No poverty,” “Zero hunger,” and “Climate action” [25].

### What Is Resilience Informatics?

Informatics has been defined as “the science of information, the practice of information processing, and the engineering of information systems” [26], although specific definitions for the field and its subfields, such as health and medical informatics, still continue to be debated [27]. Informatics tools include data science, artificial intelligence (AI), mobile health, and augmented and virtual reality. Resilience informatics (RI) is an emerging discipline concerned with harnessing informatics to materially improve and promote the ability of people, communities, and organizations to effectively cope with natural and man-made stressors [3]; for example, there is evidence in the literature that informatics tools such as medical informatics systems or informatics tools such as telemedicine and enhanced health information systems may be able to strengthen the resilience of health care facilities [28].

While modern informatics technologies can play a major role in helping people and communities develop resilience, much formative research needs to be conducted to understand the contours of this emerging discipline. The following fundamental research and development questions need to be addressed to lay a solid foundation for RI:

- For whom should we develop RI tools?
- What are the important resilience challenges to be tackled using informatics? What are the priorities?
- How can we provide privacy and security, protect sensitive data, and account for social determinants of health?
- How can we safeguard the rights of Indigenous populations and help bridge the digital divide?
- If, as seems necessary, developing resilience entails behavior change in people and communities, how can we develop tailored and personalized tools to help support behavior change?

In addition, RI systems need to be scalable and extensible while not consuming excess energy or environmental resources that could exacerbate climate change. To answer these questions, input is needed from frontline workers across diverse fields, areas of expertise, and levels of implementation in public health and resilience areas.

### The Workshop’s Purpose

On November 20, 2023, the University of Arizona hosted a workshop, titled Resilience Informatics in Public Health, with experts from a variety of relevant fields, including resilience, public health, climate, informatics, policy, and technology. These experts were primarily based in Arizona, but a few purposefully selected experts from outside of the state with relevant areas of expertise were also invited to participate and present relevant information to help set the groundwork for the workshop discussions. The purpose of the gathering was to shape the future directions and applications of the emerging discipline of RI.

A secondary purpose of the workshop was to provide networking opportunities to build connections across this field and inspire new projects in this up-and-coming area of research and development of RI tools and systems. Attendees had the opportunity to meet and network with other experts in the field whom they may not have had the chance to meet in other professional settings.

## Methods

### Recruitment

A group of 40 experts were purposefully selected and invited via email to attend the workshop. The workshop participants were from the fields of public health, medicine, weather services, informatics, environmental science, and resilience. The experts were chosen using a convenience and snowball sampling method based on whom the RI workshop leadership had developed relationships with and who seemed to be best suited to represent the desired sectors and enrich the conversations at the workshop. The institutions represented included Weill Cornell Medicine, the University of Colorado, Northern Arizona University, the Arizona Center for Rural Health, the Mel and Enid Zuckerman College of Public Health, the Arizona Institute for Resilience, and the College of Medicine-Phoenix.

### Workshop and Data Collection

During the workshop, participants listened to presentations from a few key experts to spark discussion and ideas. In between the talks, 6 groups of 3 to 7 people each were formed by workshop facilitators such that the groups contained as much diversity as possible in terms of the members’ backgrounds (field of work and discipline). Subsequently, with the help of the facilitators, the attendees reflected and collaboratively discussed answers to the questions presented in Textbox 1.

During the discussions, participants were asked to write down their ideas independently on sticky notes and compile them on flipcharts to ensure that no one participant dominated the discussion. Data saturation was not discussed because this was a 1-time event, and additional data collection was not possible after the workshop. The discussions around the key questions (Textbox 1) were systematically audio recorded, and notes were taken by students from the University of Arizona who were part of the organizing team. Workshop attendees were made aware of the audio recording and note-taking and were informed that the findings of the workshop were to be compiled into a proceedings report.

**Questions for the Resilience Informatics in Public Health workshop discussion aided by facilitators.**

**Session 1 (approximately 30 min)**

- What does resilience mean to me?
- To what do we need to be resilient?

**Session 2 (approximately 1 h)**

- What informatics tools are you using?
- What are the barriers to technological solutions? How do we address them?
- What tools do we need?
- How do we ensure equity across these solutions?

### Qualitative Analysis

**Figure 1:**
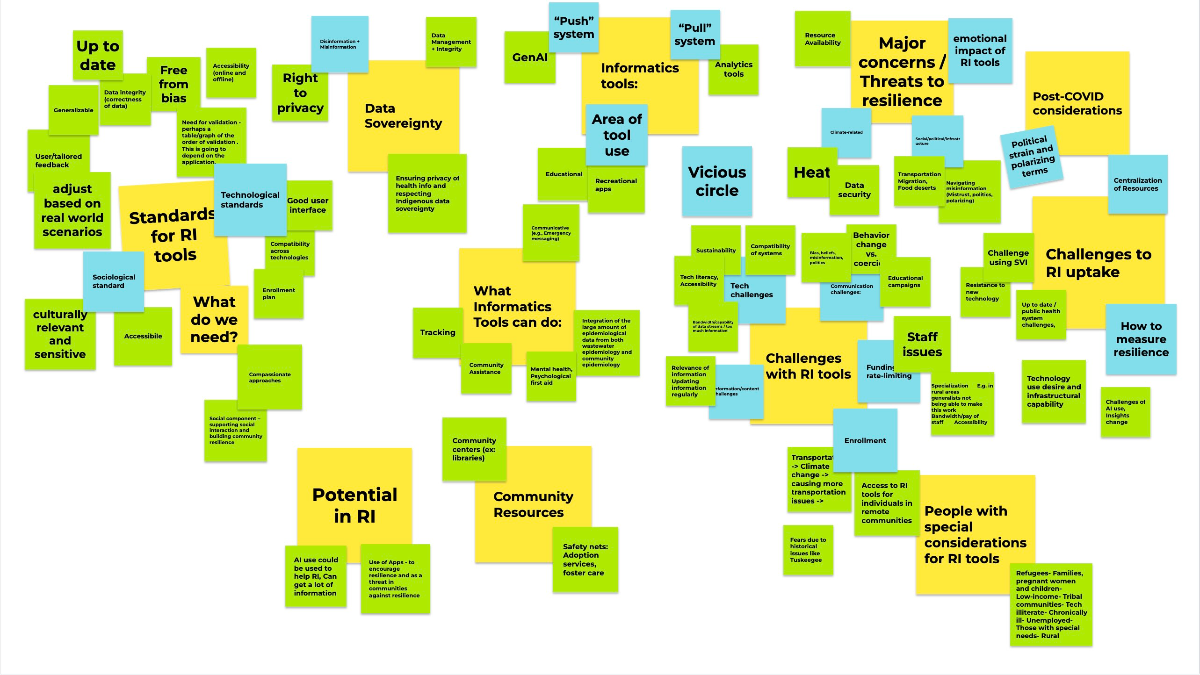
Brainstorming document for the development process of the codebook for the qualitative analysis of transcripts and notes from the Resilience Informatics in Public Health workshop.

Three University of Arizona students conducted a rapid analysis of the notes taken during the workshop to produce a report for dissemination to the greater community for awareness. Although transcripts were not sent to participants for comment, the report was shared with participants via email to solicit feedback to increase the validity of the analysis as a form of participant checking. Workshop participants were also asked to participate in the in-depth thematic analysis process. A group of 4 students and the principal investigator worked with 1 expert attendee who volunteered to help develop the codebook. The team used a primarily inductive approach in the data analysis with a phenomenological theoretical approach [29]. The analysis team members iteratively constructed their list of themes based on the transcripts and notes using standard identification techniques [30]. The themes were compiled together using a Google Jamboard and grouped to determine the final list of codes and subcodes (Figure 1).

The 4 student researchers then coded the transcripts independently. In the case that the notetakers forgot to record the discussions, notes were used in place of transcripts. The coding team also coded the notes that were taken on sticky notes by the groups during discussions to ensure that no ideas were missed. The team then worked together to finalize the first draft of the codebook with definitions and examples (Figure 1; Multimedia Appendix 1). The codebook was entered into MAXQDA (VERBI Software GmbH), and the team began coding. The first transcript was coded by all team members, and discrepancies were resolved through discussion and consultation with senior members of the team. The remaining transcripts and notes documents were coded independently by 2 coders each. Conflicts were again resolved through discussion. The codebook was adjusted through the coding process because themes may have come up inductively. After coding all documents, the coders reviewed all documents once more to ensure that nothing was missed.

The team then wrote a narrative analysis of the findings, summarizing major points that came up to help answer the workshop’s questions. The analysis write-up process was aided in part by ChatGPT (OpenAI) for certain sections in the synthesis of the summary of the information gathered from the text; these syntheses were subsequently reviewed manually to ensure accuracy, validity, and appropriate language use in the narrative. The research team used the COREQ (Consolidated Criteria for Reporting Qualitative Research) checklist to guide the reporting of the workshop findings and qualitative analysis [31].

### Facilitator Reflexivity

The facilitators of the workshop were 3 female professors and researchers with PhD degrees and experience in the fields of epidemiology, environmental health, climate, and resilience. One of the facilitators is a widely recognized infectious disease epidemiologist with practical experience in public health practice at a health department level and a focus on the environmental determinants of vector-borne disease transmission and control. Another facilitator is an assistant professor at the Mel and Enid Zuckerman College of Public Health with a focus on building public health capacity to address “complex” public health challenges through developing decision-support tools, enhancing science communication and translation, and integrating a health and equity lens into adaptation planning. At the time of the workshop, she was leading the Arizona Department of Health Services–Centers for Disease Control and Prevention COVID Health Disparities Initiative at the Arizona Center for Rural Health aimed at mobilizing partnerships to advance health equity and address social determinants of health related to COVID-19 health disparities among underserved populations considered high risk. Finally, the last facilitator is an experienced research professor with the Lyda Hill Institute for Human Resilience, faculty at the University of Colorado Denver and the Mel and Enid Zuckerman College of Public Health, a guest researcher with the Centers for Disease Control and Prevention, and an affiliate scientist at the National Center for Atmospheric Research in Boulder, Colorado. She focuses on the intersection of weather, climate, and health, with an emphasis on vector-borne diseases.

Workshop participants were primarily invited because they had a relationship with at least 1 of the facilitators in their line of work related to public health and resilience. The participants were familiar with the researchers and their public health interventions, which have benefited the state of Arizona, especially in relation to the COVID-19 pandemic.

### Ethical Considerations

The RI workshop team reviewed the criteria for determining human subject research and concluded that the study protocol did not meet the criteria, classifying it as nonhuman subject research. The study team took all appropriate precautions to maintain data confidentiality. Participants were made aware that information collected from the conversations would be compiled in a report and manuscript for publication and dissemination of the workshop insights. Transcripts and quotes were deidentified to further protect participant identities as a precautionary measure. No compensation was offered, although travel, food, and accommodation reimbursements were provided for those who requested assistance to attend the workshop.

## Results

### Workshop Attendees

Of the 40 experts invited, 27 (68%) participated in the workshop at the Health Sciences Innovations Building at the University of Arizona. Of these 27 experts, 21 (78%) were from public health–related fields (n=5, 24% with a concentration in epidemiology; n=5, 24% with a concentration in community outreach; and n=3, 14% with a concentration in medicine). Of the 27 experts, 8 (30%) worked in the field of resilience, and 7 (26%) had a concentration in a field of technology such as data science (n=4, 57%). Finally, of the 27 experts, 3 (11%) had extensive experience in emergency management or preparedness, 1 (4%) in environmental science, and 2 (7%) in meteorology.

The organizing team present included the 3 facilitators, 1 principal investigator, and 8 students from relevant fields who helped with logistics, note-taking, and audio recording of conversations.

The largest number of participants were from academia, mostly affiliated with the University of Arizona (19/27, 70%), followed by Arizona State University (3/27, 11%), and specially invited attendees from academic institutions outside of Arizona (University of Colorado: 1/27, 4%; and Weill Cornell Medicine: 1/27, 4%). Of the 27 attendees, 2 (7%) came from the Arizona Department of Health Services, while 3 (11%) were from county-level health departments (Pima: n=1, 33%; Coconino: n=1, 33%; and Maricopa: n=1, 33%); furthermore, 3 (11%) participants came from organizations representing American Indian groups (Salt River Pima-Maricopa Indian Community: n=1, 4%; and Arizona Advisory Council on Indian Health Care: n=2, 7%), 2 (7%) were from the National Oceanic and Atmospheric Administration and National Weather Service, 1 (4%) was the chief executive officer and founder of a mobile health organization (Wehealth), and 1 (4%) was from a statewide community health worker organization (Arizona Community Health Workers Association).

### Codes

**Figure 2:**
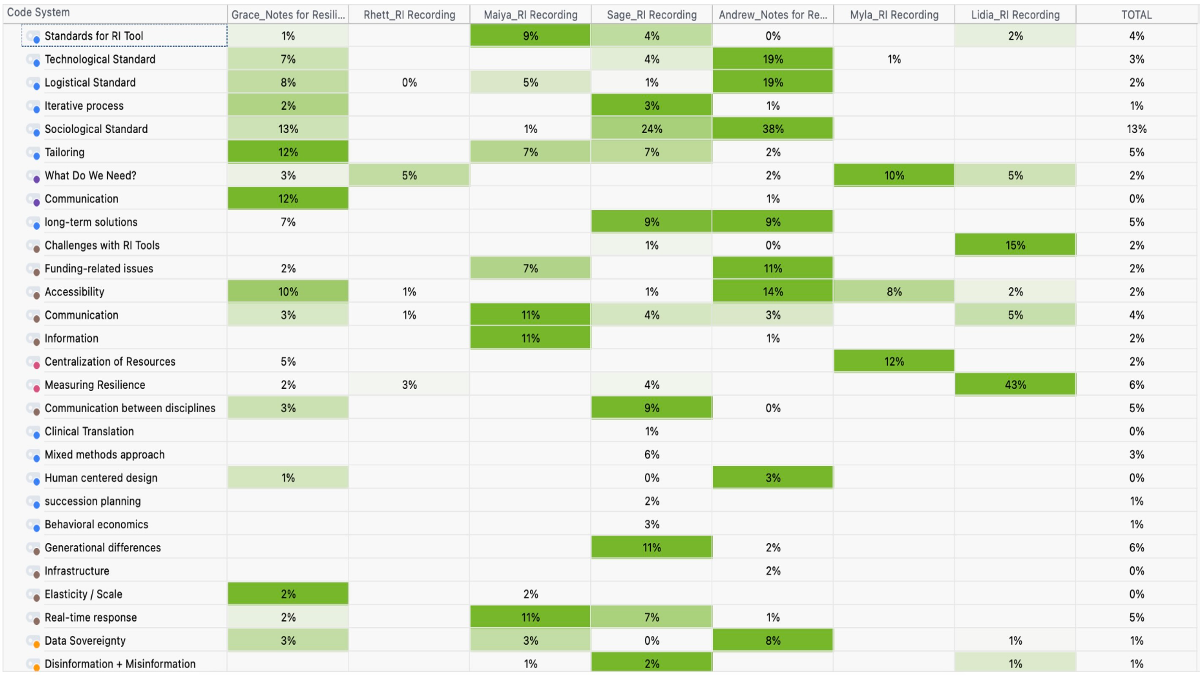
Frequency of codes in the transcript and notes documents included in qualitative analysis from MAXQDA.

The codes that were identified during the qualitative analysis included the potential in RI, threats to resilience, people with special considerations, challenges with RI, standards for RI tools (technological, sociological, and logistical), and data sovereignty, among others (Multimedia Appendix 1; Figure 2). The major findings associated with the codes are included in the following subsections.

#### Potential in RI

RI tools were mentioned in many of the conversations. Specific examples included the 4 tools highlighted during the expert presentations at the event—Pilas, AZCOVIDTXT, AZX, and Wehealth—as well as ≥200 essential tools for public health data monitoring (Textbox 2).

RI tools were also used for educational purposes such as community education, health care worker training and simulation tools. Such tools were also mentioned as having a multitude of different purposes and functions (Textbox 3).

**List of key existing resilience informatics tool examples mentioned by participants in the workshop.**

- Organization assessments
- Quality assurance tools
- Heat maps
- Telemapping tools
- Mental health screening
- The 988 public helpline for mental health

**Purposes of key existing resilience informatics tools.**

- Creating clinical guidelines with community perspective and provider perspective
- Recording membership sign-ups
- Listing contact information for outreach in emergencies
- Taking raw data and putting it into a usable format
- Digesting complex information, summarizing relevant information, and then pushing it to the relevant audience
- Recording how many people have enrolled in a course
- Evaluating a course or intervention
- Measuring the intensity of reactions from the public
- Surveying the prevalence of infectious diseases or chronic illnesses
- Acting as a decision tool for gauging and assessing data
- Projecting the impact of an intervention

Finally, emerging technologies such as generative AI hold the potential to enhance the impact of RI, as highlighted by a participant:

So this is actually a really hot area of research for generative AI. And you have to look at it a little bit differently than what you see on the surface. But what generative AI is, you can feed it a corpus of knowledge. And it builds a giant tree out of that knowledge. And so when you ask a specific question, it’ll try to navigate that part of the tree and find those things...I mean, there’s reasons to be skeptical because basically the ChatGPT systems that we know have gotten their information from all over the internet. There’s some really bad information. But there are other ways where, for example, Maricopa County could put all of your policies, plans, procedures and every...Build your own corpus of knowledge and use the same generative AI algorithms so that you could ask a question like, “How do I answer this question?” And so then it’s taking your internal information, sort of trying to like capture your brain and everyone else’s brain and all of the documents and you can produce stuff.

Participant from Arizona State University Knowledge Enterprise

In summary, RI tools have the potential to span a wide range of functions. While many RI tools are already available, there remain numerous possible areas for further expansion.

#### Threats to Resilience

Resilience faces challenges across various health sectors, which are often intersecting and complex. Protecting the resilience of communities and ensuring their health and well-being begins with understanding these threats and how individual communities are impacted.

Health care access was one of the threats to resilience mentioned at the workshop. The cost of health care alone makes it inaccessible to many groups of people or, at the very least, a burden to individuals who are barely able to afford health care. However, threats to health care access extend beyond cost because the geographic location of health services also plays a critical role. Our participants also mentioned that within health care services, workforce strain can pose a threat to public health. Health practitioners’ mental health was also mentioned as having a direct impact on the quality of treatment that patients receive. Overworked health care professionals introduce the potential for practitioner mistakes or lead to a shortage of health care professionals in the community, as noted by a participant:

But then you take into account that 30% of the public health workforce left the profession during COVID. And one of the biggest challenges in health departments right now is retention, not just retention of people, retention of historical knowledge and community understanding. I mean, of this idea. And I’m like, “Yes.” But it really sucks when you have a room full of people who don’t really know, understand and haven’t worked, been on the ground during the pandemic, haven’t done these things, don’t know the community.

Participant from Arizona Advisory Council on Indian Health Care

*Inadequate infrastructure* was mentioned by our participants as a large threat with respect to the centralization of resources and transportation access, as previously discussed with health care access. However, the threat of infrastructure extends beyond these areas, encompassing the housing crisis, internet access, and utilities. The centralization of resources and transportation services is necessary to protect the health of communities, as discussed before, in terms of the ability to access health services. However, beyond health care, a lack of transportation services and decentralized resources continue to threaten resilience because transportation is crucial for individuals in food deserts to be able to reach grocery stores and for those in areas of job shortages to reach work. Housing accessibility and affordability were also mentioned during the workshop because housing prices have increased in the past few years in Arizona.

In the case of water, communities rely on having access to safe, running water for consumption and bathing. This important resource, especially in the desert environment of Arizona, has not always been adequately protected during droughts and instances of contaminated water supply, as mentioned by a participant:

So, what has happened in a lot of the orchards, like the nut orchards, have moved from California to the Willcox area [in Arizona] to pump the water out because they don’t have any restrictions. And so basically, they’re sucking it dry. So, the concern is, there won’t be any water in the future, 50 years from now, in that area.

Participant in environmental science field at the University of Arizona

Internet access was mentioned as being of great importance for protecting health as well because much health-related information and many health services are available on the web. Being able to view this health information or find health services through web searches is important for individuals to be able to protect their health.

*Large-scale events* such as the ongoing climate crisis were mentioned during the workshop as one of the greatest threats to resilience. Climate change has negatively impacted water resources, raised temperatures, and increased the prevalence of infectious diseases. Furthermore, modern infrastructure is not equipped to withstand the challenges that extreme weather events pose, leaving communities more at risk of being devastated by such an event. One participant stated as follows:

How, for me, is its emergency response, but it’s also how to prevent and mitigate issues with climate change. And we’re getting into it with the hottest heat on record in the city of Phoenix. I’m trying to get more involved with getting areas that don’t have cooling centers, doesn’t have things like that, like the tribal nations...Outlying areas of, even in the Phoenix area, doesn’t have cooling centers...So, we see a big phase for central Phoenix and getting in some rural areas, but when you start going outside the Phoenix area or into tribal nations, there’s nothing to help them recover or even get supplies. So, to me, that’s a big issue. I live in Queen Creek, so even looking at my own town, they have a station. Yeah, it’s the fire station. You drop off the water, but you have no cooling stations for the homeless or individuals or even the reservations.

Participant from the Arizona Coalition for Healthcare Emergency Response

The climate crisis has become even more difficult to address due to its politicization.

*Political strain* was mentioned at the workshop as another threat to the resilience of communities in working against the polarization of health concerns. Participants mentioned that during the COVID-19 pandemic, the communication of accurate information proved difficult because the public was inundated with mass amounts of misinformation and disinformation. One participant stated as follows:

Politics can modify how we approach public health disasters.

Participant from a county health department

*Communication* emerged as an individual threat to the resilience of communities because approaches to addressing the COVID-19 pandemic were disjointed and limited as a result, according to our workshop participants. There were disparities in information between clinicians and public health practitioners who lacked the means to effectively engage with each other. The inability to properly communicate exacerbated the issue of trust in information and the prevalence of misinformation. Creating adaptable communication systems can pose difficulty in the field of public health, as mentioned by a workshop participant:

I think uncertainty too. There’s a lot of the things we’re talking about happening in the future. I think especially when we talk about extreme heat and things like that, or even other things like how AI is going to come into play. There’s just a lot of uncertainty of what’s going to happen. I think that really heavily influences the way people respond to information. How do you communicate something that, things that are changing, things that are uncertain, and how do you make decisions in an uncertain environment, the burden that puts on a household person trying to plan financially or somebody just mentally the load, or even a city planner trying to figure out what’s the best decision?

Participant from Arizona State University Knowledge Enterprise

Issues related to access to *resources* also emerged as a potential threat to resilience for the communities our participants serve. A lack of financial resources limits what the community can afford to support its health, as noted by a participant:

And it’s for our community organizations so that they can leverage that piece to apply funding, to ensure there’s more programs within the services, within the communities they serve. Because we can’t do everything. We don’t have the capacity.

Participant from a county health department

Other resource-related issues mentioned by participants included food supply and air.

Several *social issues* were mentioned during the workshop that directly work as threats to resilience, with public health professionals facing concerns surrounding food insecurity, the housing crisis, transportation, migration, racism, gentrification, war and politics, cultural sensitivity, the cost of living, employment security, and the education system. One participant stated as follows:

Yeah. I think distrust is more just the fact that if we need to be resilient, we need to trust each other and we don’t anymore. And so that’s a thing we need to be aware of. How do we build trust in communities? And the disinformation is more about are we just getting the right information or not? That’s why I meant trust between communities.

Participant who is a software development professional and chief executive officer of a mobile health organization

*Mental health* was a concern raised by several participants; for instance, aging populations were mentioned as being vulnerable to the empty nest syndrome and isolation issues. In addition, substance misuse was directly related to mental health (eg, the growing prevalence of drugs such as fentanyl). Concerns surrounding the lack of empathy and social capacity were discussed in depth in 1 group at the workshop. The lack of empathy in American culture was mentioned as possibly stemming from the colonial mindset focused on individualism. By contrast, social capacity was identified as an issue due to the ever-growing role of mobile phones and social media today, especially among younger generations. Mindful interventions were mentioned as having the potential to address these concerns and help ensure that populations remain socially capable. One participant stated as follows:

It’s compassion fatigue or something. You get so much information because you’re connected to the internet, you just get bombarded every day.

Participant from a county health department

#### People With Special Considerations

A recurrent theme that emerged during the workshop discussions was that certain communities may face greater challenges to resilience than others, as mentioned by a participant:

No one would choose not to have running water.

Participant from a county health department

It was apparent in many of the discussions that some communities lack basic resources such as consistent access to water. Indigenous communities, asylum seekers, the aging and older adult populations, people with disabilities, non–English-speaking communities, and other groups considered marginalized often face unique challenges to resilience that merit special considerations when considering the use of RI tools.

Asylum seekers and refugees were mentioned as having challenges related to privacy and access to resilience tools because they are vulnerable to exploitation and often face barriers to legal recourse. The aging and older adult populations, people with disabilities, non–English-speaking communities, and other groups considered marginalized were also mentioned as needing special consideration when designing RI tools (eg, considering translation, accessibility, and other tailoring approaches to fit their needs).

#### Challenges With RI Tools

Some workshop participants mentioned that RI tools face challenges of usability that must be understood and addressed to maximize the positive impact of the tools in improving community resilience while also preventing possible adverse effects.

Accessibility is the first of these challenges. RI tools can be limited in impact based on the size of the audience they can reach and effectively assist. Tools should be tailored to the community being served and designed with end users in mind. Elasticity and scalability were mentioned as issues that should be at the forefront of RI tool design considerations so that the tools may serve the greatest number of communities, which can vary in size, geography, and so on. Accessibility concerns further include issues of resource centralization, language barriers, technology literacy, and related considerations. One participant remarked as follows:

So, one thing too, I would say the integration of social determinants of health in city planning. So general plans as well as emergency response plans. One of the things we learned during COVID is a lot of our emergency response plans really weren’t built to address supporting diverse populations, multilanguage speakers, all of these other things. And so being able to go in and make those the norm now.

Participant from a county health department

Real-time response was mentioned as being especially desirable in RI tools. Between collecting and analyzing data and publishing the results, the process can be all too slow to be able to properly respond to fast-moving issues that may threaten resilience, such as a pandemic. RI tools must be able to quickly respond in times of crisis to be able to limit the amount of people who are affected by the crisis, as highlighted by a participant:

Informatics can assist me in finding what that trigger point is, but it can’t assist me in actual response. It’s too late. Once it’s already happening, now I’m in the door. I’m working with a problem and actually implementing things. Informatics takes a backseat; they don’t care anymore. But I want informatics to inform that response. That would be very valuable to tell me, “Hey, you’re done. You can stop now. You can turn it off.” That would be nice. But I don’t know how informatics can assist in the response phase or the recovery phase.

Participant from a county health department

Funding was mentioned as being a particular challenge for existing public health and resilience-building initiatives. RI tools and projects must be able to secure funding, such as through grants, to establish and sustain the tool. An example given by a participant concerned the difficulty of fitting grant expectations. If an RI project does not fit expectations, adjustments will need to be made to maintain funding, and the project’s efficiency may suffer. Funding is necessary for developing RI tools, but it is also a potential threat if the tool does appeal to funders. A participant stated as follows:

Make recommendations to other departments and divisions within the health department to say, “Hey, we need to allocate resources here. Here’s what we’re hearing.” And to your point, we’re listening to people’s individual stories. We’re also looking at data as a whole. And the reality is, we’ve tried to push a lot of these dashboards and a lot of these things to the general public...They’re not really interested in a lot of that unless it’s very easy to use and speaks directly to them. So again, my job is that, “Okay, that’s not working. And how can I frame it in a way that’s really going to focus on that individual so they can make an important decision themselves?” So a lot of what we’ve done is really just change the way we present data.

Participant from a county health department

RI tools are also tasked with the challenge of measuring resilience, especially across the various fields that contribute to resilience research; for instance, resilience must be defined in a standard way, and units of measurement may need to be standardized according to the definition. A group of participants had the following discussion:

Speaker 6: Do you guys think that’s a problem, the way that we measure their resilience, and solve problems in this case? So the thing that we should think about how we are measuring this resilience about other things right here, I don’t know if we have the right tools right now to do that. The resilience measurement tools.

Speaker 9: And then the definition of resilience, which alters person to person, agency to agency.

Speaker 6: Yeah, that’s true.

Speaker 9: Race, ethnicity, there’s so many implications, things that affect that. So I always look at it as you have to define what the meaning is first in order to measure it. Otherwise, its positive outcomes, just how you go about it. What do you define as success in the situation? Again, that’s always going to alter and shift.

Speaker 8: Or how do you weigh different? If you can agree that resilience is sort of a combination of metrics that we already account for, okay, so we’re counting. We’re trying to measure things like food security and vulnerability, heat and different mental health metrics. But then you try to combine that into some sort of measurement of resilience. It’s like, “Well, how do I weigh in some sort of statistical way, the weight of my mental health versus my heat resilience, which are all...”

Later, in the same group, a participant stated as follows:

We spent all this time trying to measure the wrong things, like risk. Risk is great. Let’s take probability times consequence and its risk. And then we’ve got everything solved and we’re measuring the wrong things. So at least now that we can talk about resiliency and all of the constructs that come with it, you package it up and you’ve got to talk about social cohesion and adaptivity and vulnerability, and then you sprinkle in some of that probability and consequence stuff. But that’s so reductive that you miss the point of what resilience is, which it’s an intended positive outcome about how we can work together and have shared goals that are different than individual goals. But you have to have all of that. So, I think it’s a really positive thing for results is hard to measure, and you have to roll around in the mud with a pig for a while in order to put some thoughts together. Yeah, it is refreshing.

Participant from Arizona State University Knowledge Enterprise

#### Technological Challenges With RI Tools

Several challenges that are more technological were also mentioned by many participants; for instance, infrastructure issues such as broadband access are problematic for the effectiveness of RI interventions, which often rely heavily on the presence, functioning, and quality of technological tools. At times, government intervention may aid in increasing the necessary infrastructure; for example, some government programs could help to provide free Wi-Fi or expand hotspots. Rural communities were mentioned as being especially vulnerable to such issues, where there may be higher turnover in technology. In this case, low-cost smartphones may be needed to support these communities, or perhaps a community-operated mobile phone provider could be enlisted to help increase mobile phone coverage and access.

#### Communication

RI communication faces a spectrum of barriers and facilitators, prominently highlighted by the necessity for 2-way communication. This involves ensuring a cohesive message and fostering a shared understanding among stakeholders. However, intergenerational disparities present challenges because differing communication preferences and styles may hinder effective dialogue. Establishing rapport amid these differences can prove arduous, as illustrated by the struggle to navigate technological nuances and preferences, such as the preference for traditional email over newer communication methods.

Moreover, the landscape of informatics communication is marked by silos, where various disciplines converge, including developers, computer scientists, behavioral economists, public health practitioners, and social scientists. This interdisciplinary nature can be both a boon and a bane. While it offers diverse perspectives, it also necessitates concerted efforts to bridge disciplinary divides. One expert at the workshop asked the following question:

What about in nonemergent situations? Nonemergent public health situations? How can that gap the bridge? And I’m talking about between the academics in between the field of public health and then between the clinical side? That’s my question.

Participant from Arizona Advisory Council on Indian Health Care

In addition, the gap between the clinical and public health realms underscores the need for enhanced collaboration and communication strategies to align goals and priorities effectively. Overcoming these barriers requires deliberate efforts to foster a culture of inclusivity and open communication across sectors and disciplines.

#### Components of an RI Project

Several different essential components of a successful RI tool or project were mentioned during many of the conversations (Textbox 4).

**Key considerations and essential components when designing a resilience informatics (RI) intervention.**

- Systems and infrastructure to process data (broadband access)
- Data integration hub and intelligence subsystem for information sharing
- Anomaly finder (could be an artificial intelligence [AI] function with data search)
- AI to digest complex information, summarize relevant information, and then push it to the relevant audience
- Centralized hotline or integration to allow individuals to have a resource to call
- Bidirectional flow (receive community input)
- Equity assessment (part of before, during, and after evaluations)
- Human elements and user tests
- Coalition oversight to have a big-picture view and report back
- Communication component to hit all different networks

#### Standards for RI Tools

##### Overview

A major theme that emerged from the RI workshop discussions was the need for standards or guidelines to help guide the design and implementation of RI tools in the future. Through an analysis of participant conversations, a list of standards for RI tools was developed based on the characteristics presented as ideal for RI tools. While it is recognized that this list may not be comprehensive and that some of the standards may apply to some but not all tools, it may serve as a starting point for future iterations of RI tool standard development. The standards have been grouped into 3 categories based on the nature of the characteristics in question: technological, logistical, and sociological.

##### Technological Standards for RI Tools

Technological standards were mentioned in many of the discussions. These standards may be helpful for software developers, statisticians, and other technical professionals involved in RI projects.

###### RI Tools Should Be Up to Date

Similar to other forms of technology, RI tools must be regularly maintained and updated to ensure continued accessibility and usefulness, according to our participants. RI practitioners must also put into place standards for data used by RI tools. This may entail connecting multiple resources to get a broader scope of data, training AI tools by using data from diverse populations to remove bias, establishing standards to ensure the accuracy of data, and using validated tools and objective measurements when implementing RI.

###### Data Must Be Accessible

Another concern regarding RI tools mentioned in the expert discussions at the workshop was accessibility: data should be designed to be accessible (following accessibility guidelines) while still being protected. Tiered access to data (eg, nonpublic facing or anonymized reports to preserve the privacy and security of data) should be implemented. There should also be regulations in place related to the sharing of private corporate data, such as the number of COVID-19 test kits sold.

###### RI Tools Should Be Interoperable

Furthermore, RI tools need to be optimized for all major platforms to ensure interoperability across different technologies. This could involve creating versions of RI tools for Apple and Microsoft operating systems, among others.

###### Data Sovereignty and Privacy Should Be Prioritized

Finally, protecting data sovereignty and privacy must be of utmost importance for RI tool developers and users. This especially applies to the use of RI tools with populations who may be particularly susceptible to abuse or exploitation by bad actors. Data-sharing agreements can be beneficial tools for parties collaborating on resilience-related public health projects with RI tools; however, these agreements must explicitly be written to protect the sovereignty, ownership, and privacy of the data collected and shared.

##### Logistical Standards for RI Tools

Another type of RI tool standard that emerged from the discussions among workshop attendees focused on logistical characteristics. These are more related to the planning, organization, design, and practical aspects of implementing or rolling out the tool, rather than the technological or sociological aspects of the tool or RI project.

###### RI Tools Require Thoughtful Design

It was mentioned that when designing RI tools or interventions, it is important to use a systematic approach, following step-by-step procedures. These procedures should include data collection and performance evaluation to ensure continued monitoring and evaluation of the project, ensuring that objectives are met and that the necessary information is available to demonstrate the effectiveness of the intervention. Examples of disciplines or approaches from which RI tools could draw best practices include human-centered design, mixed methods research, and clinical translational science. The acceptability of the desired behavior that the RI tool may be promoting should always be considered as well in the design and implementation of the project. The design could additionally include an implementation model, considering infrastructure, service, providers, users involved, and so on.

###### The Process of RI Tool Development Should Be Iterative

The workshop discussions highlighted the need for continual community testing and evaluation. One participant stated as follows:

We’re always reinventing.

Moderator from the University of Colorado

###### RI Tools Need to Have Real-Time Responses

RI tools should work in real time and be able to pivot and adapt quickly to fit the needs of end users. Rapid and clear information should be conveyed in a simple report afterward as well.

###### RI Tools Need to Have Multiple-Layer Solutions

RI tools should function at a variety of levels to meet the needs of the communities they serve; for example, an ideal RI tool should consider all channels that the end users may use: SMS text messaging, apps, social media, and so on.

###### RI Tools Require Funding

While perhaps evident, RI tools need funding to be most effective, and this was emphasized many times at the workshop. In the planning and design stages, it is important to consider where these funds will be coming from to make sure that the tool is most effective and sustainable: grants, sales, and so on. Multiple funding streams may be best to consider, should it be possible. In addition, funding is important to consider when budgeting. One participant stated as follows:

How can I give our community partners something that they can turn around and use as leverage for money, resources, whatever?

Participant from a county health department

##### Sociological Standards for RI Tools

The following sociological standards for RI tools were identified at the workshop.

###### Focus on Community-Identified Needs First

Our participants mentioned that RI tools should prioritize populations with the greatest needs within the communities being served. To do so, stakeholder engagement in critical conversations about design and intervention implementation is crucial.

###### Build Trust: “Partnerships Are Key!”

Upfront community engagement is key to RI tool success. Community leaders and partners, particularly gatekeepers to the communities, need to be engaged in the RI tool from the outset. Community members should be additionally involved in decision-making surrounding resource allocation if this is relevant to the RI tool or project. Successful community engagements are characterized by humility; in other words, our experts mentioned that it is important to be upfront about what you do or do not know. Along the same lines, community values must be recognized early on to ensure successful community engagement, such as transparency, humility, and so on. To identify these values and develop community partnerships, we must listen to groups (eg, tribal communities), understand and learn from others, and recognize that all people have important information, no matter their education level or background. Cultural humility and responsibility as well as community-based participatory research methods may provide important lessons from which RI tool developers and implementers can gain a better understanding of successful community engagement and cultural value identification.

###### Integrate Social Determinants of Health

The RI workshop participants mentioned how important it is to integrate social determinants of health, such as income, education level, and health care access, into the design, implementation, and evaluation of the RI tool. These factors are crucial when considering public health outcomes because they can influence and potentially confound the tool’s impacts.

###### Long-Term Solutions and Sustainability Should Be the Goal

The RI tool must have a long-term vision and sustainability as the goal to most effectively build resilience in communities. One participant stated as follows:

Systemic change takes time, and it builds, and you’ve got to take things and use things to your advantage.

Participant from the Arizona Department of Health Services

Along these lines, the RI project will need a way to follow up with participants over time to continually evaluate and assess the long-term impacts of the RI intervention.

###### Tailor the Intervention

Tailoring was a strong theme in many of the conversations that experts had at the workshop. Each RI tool must be tailored to the specific population of interest. One participant stated as follows:

So you know what’s dangerous about that idea is that it’s measuring urban capability against rural ability, because you’re talking to the one of two people in the entire county that is thinking about public health weather. There is no one else.

Participant from a county health department

RI tool designers must additionally contextualize the problem not only by population but also by the specific event as well.

Participants also mentioned that intergenerational solutions (eg, physical community spaces) are important when developing RI tool interventions. Language must always be considered—not only in its conventional sense but also in terms of vernacular expressions and subgroup dialects—to increase understanding when using RI tools to communicate with different communities. Cultural representation must also be considered when tailoring interventions, considering differences in emergency response language and acronyms and literacy levels. One participant warned against reliance on AI translation, although it seems that AI tools are evolving at a rapid rate and may improve translation capabilities over time. In the meantime, it may be crucial for most communities to either rely on manual translation with the help of local community members or, at the very least, double-check AI-generated translations through manual review. Multimedia and multimodal strategies were suggested as being important to address language barriers or varying literacy levels. Differences in technology accessibility must also be acknowledged and taken into consideration in RI tool design and implementation. To be most inclusive, low-tech or even no-tech alternatives may need to be integrated into the design. One participant remarked as follows:

I always say, “It’s the generation of someone who prefers a phone call than a text.”

Participant from the cooperative extension office, affiliated with the University of Arizona

Finally, it was emphasized that it is important for all RI tools to be inclusive for people with disabilities.

###### Equity Must Be Prioritized

During the workshop, participants were asked to answer a question about how equity would be prioritized in the RI tools; for instance, it is important to collect data to evaluate equity within the RI intervention, such as documenting equity awareness and feedback. Another way to prioritize equity in the RI project could be by involving people from the community in project roles and paying them equitably. Assessing policies and tools (eg, AI) for discriminatory impacts may be an equitable goal or activity for an RI project. Finally, it was recognized in a conversation that the Culturally and Linguistically Appropriate Services standards are an existing guideline that may be useful for RI tool developers to reference and use to increase equitable outcomes.

### Issues Related to Data Use

Two main issues emerged in the discussions regarding data use (Textbox 5).

**Issues regarding data use and illustrative quotes.**

**Data are not always presented in an appealing or accessible way for practitioners**

“The informatics can inform our response, but we might be just focusing so heavily on the data that the practitioners out in the field start going, yeah, it’s a wildfire, what do you want me to do about it? It’s going to burn. It’s going to burn that long.” [Participant from a county health department]

**The right information must be there for somebody to grab it**

“I used to develop a lot of software...and we used to do pharmacy applications and our user group would say, ‘We need these 20 data fields.’ We go, ‘Yeah, that makes sense. Okay, we’ll put the 20 data fields in.’ Three years later we go back, two of them are used. Now did that mean the other 18 weren’t important or shouldn’t have been used? It probably meant that there was this really subset that needed those data fields to make better decisions. But if you looked at it from utilization of the data field, you would say, ‘Well, just get rid of them, we really don’t need them.’” [Participant from a county health department]

### Indigenous Data Sovereignty

Indigenous data sovereignty was a key theme that emerged from the guest presentation from the Salt River Pima-Maricopa Indian Community and the discussions among the workshop attendees. Indigenous data sovereignty can be defined as “the right of Indigenous Peoples to govern how data from or about them is collected, accessed, used, stored, and disposed of” [32]. Culturally centered sovereignty requires a conscious effort by public health officials, researchers, policy makers, and RI practitioners alike. Explicit protections and acknowledgments of Indigenous ownership of data and the rights surrounding these data must be put into place before RI tools are deployed in Indigenous communities. This involves an acknowledgment of cultural practices, the political sovereignty of Indigenous nations, collective knowledge and shared wisdom, and the relationship between Indigenous peoples and the land.

Martinez outlined 4 principles that must be considered for Indigenous governance known as the “CARE principles”: collective benefit, authority to control, responsibility, and ethics. Following these principles is a minimum standard for public health work in Indigenous communities, particularly with respect to sensitive health data collected about Indigenous peoples [33].

## Discussion

### Principal Findings

In summary, the RI workshop experts confirmed the need for tools that translate technical knowledge into something useful for the community. These tools must be low cost or free, accessible (easy to use), available on any platform (especially mobile phones), and simple in design; not require special domain knowledge; and be connected, bidirectional, responsive in real time, and respectful of data sovereignty. Technological and multimodal alternatives tailored for different groups with varying accessibility needs are crucial for the success of RI tools. The end goals of these RI tools are sharing resources, effecting behavior change, and advancing health equity.

### Comparison to Existing Literature

Some of the findings from our workshop resonate with those in similar studies in the literature; for instance, our attendees confirmed that informatics tools have the potential to help build resilience by improving the capacity for households to respond to and make effective decisions during disasters, as well as through improving social capital [34]. Another finding that resonates with existing literature is that there is a need for a structured guide for the development of informatics tools [35]. Other studies have discussed logistical issues such as data quality and difficulties related to collecting data being obstacles to the success of health informatics projects in addressing pandemics [36]. In addition, similar studies on the COVID-19 pandemic have noted that human factors, ethics, data privacy, and the diversity of participants are vital to consider in the implementation of medical informatics tools [37]. Another study emphasized the iterative nature of informatics tools, necessitating continuous evaluation and reformulation for success [38]. This paper presents a few initial ideas that could be used to begin developing a checklist or framework of standards for future RI tools. Finally, it was demonstrated in this summary from our workshop that RI, and health informatics in general, encompasses a very wide range of functions and has the potential to help make health care services more resilient in the face of climate change impacts, similar to findings from other studies on health informatics [39].

It is worth noting that our workshop did not include much explicit discussion about big data and its potential, which would be an important area to explore in the future, especially considering the potential it has shown in building resilience in other sectors [40,41]. Another important issue that did not come up, despite some conversation about the positive impacts that informatics may have on reducing workforce strain, was the concern that informatics may impact jobs in health care and resilience-adjacent sectors if not introduced with care [42]. Other concerns include the fact that while these tools may be emerging, the workforce to implement them may not be readily available [43]. This topic has been explored in the literature and has been a concern with many other major technological breakthroughs and should be explored further to preemptively avoid any major impacts in restructuring the workforce to ensure that new informatics tools do not have large negative impacts [42].

### Strengths and Limitations

This workshop included a wide range of participants from different fields, with each being an expert in their respective area, thus lending credibility and wide-reaching relevance to the findings. A limitation of this workshop is that the findings may be limited primarily to Arizona because most participants were from the state. Nonetheless, it was recognized that many of the discussions were relevant to contexts outside of Arizona as well. In addition, because this was not a qualitative study with a systematic approach to data collection, the findings may not be comprehensive, and future research should be conducted to ensure that a saturation of ideas is reached to gain a fuller understanding of the reach and potential of this field.

### Future Directions

Future research questions to explore that emerged from the workshop discussions included the following:

- How do you physically change the environment to protect against infectious disease?
- How do we leverage community knowledge? What is the compensation for this?
- How do we teach empathy?
- What standards are necessary for RI tools to be most effective at addressing public health and climate change–related issues?

It is important for future research in this area to adopt mixed methods and be interdisciplinary, human centered, and community based. The research should ensure that equity considerations are at the forefront.

A follow-up workshop was held in June 2024 in Tucson, Arizona, to present the findings and work toward developing solutions to resilience and public health problems together with the same experts from this initial workshop. For future directions in the field of RI developments, it would be helpful to have more focused discussions with clear outputs such as implementation guidelines or designs of RI tools to put into practice. It is important to continue to build relationships with communities, stay engaged and curious about different cultures, and learn from others.

### Conclusions

The RI workshop described in this paper has laid a critical foundation for advancing the development and application of RI tools, especially in the context of public health and climate change. By emphasizing the need for accessible, user-friendly, and equitable technologies that can effectively bridge technical knowledge and community needs, this workshop has underscored the importance of multidisciplinary and community-centered approaches. The discussions highlighted key areas for future research, including physically transforming environments to mitigate disease risk, leveraging community knowledge, and developing empathetic and culturally sensitive tools. With a follow-up workshop planned, the next steps involve moving from discussion to actionable outcomes, focusing on the creation of implementation guidelines and tool designs that prioritize health equity and resilience. In conclusion, continued collaboration between experts, communities, and interdisciplinary teams is essential to realizing the full potential of RI in addressing public health challenges in an era of rapid global change.

### Data Availability

The datasets generated during and analyzed during this study are not publicly available to protect the privacy of our participants but are available from the corresponding author on reasonable request.

## Appendix

Multimedia Appendix 1 Codebook with definitions and examples used for qualitative coding and analysis.

## Abbreviations

AI: artificial intelligence
COREQ: Consolidated Criteria for Reporting Qualitative Research
RI: resilience informatics
