# Supplementary material for: Resilience Informatics in Public Health: Qualitative Analysis of Conference Proceedings
Source: JMIR Form Res. 2025 Jan 16;9:e63217. doi: 10.2196/63217 (PMC11783028; doi:10.2196/63217)
Supplement: Multimedia Appendix 1 [file formative_v9i1e63217_app1.docx]

Appendix 1. Codebook with definitions and examples used for qualitative coding and analysis

| **Theme** | **Sub theme** | **Definition** | **Example** |
| --- | --- | --- | --- |
| Standards for RI tools |  | *The qualities that all RI tools should ideally have.* | *Up to date* |
|  | Technological standard | *A quality of an RI tool that is related to a technological capacity* | *Validated, accessible on and offline, good user interface* |
|  | Logistical standard | *A quality of an RI tool that is related to logistics and not to something that is more technological in nature* | *Accessible, adjusts based on real world scenarios, has enrollment plan* |
|  | Sociological standard | *A quality of an RI tool that is related to sociological aspects like culture, language, social interactions, etc.* | *Culturally relevant, generalizable,* |
| What do we need? |  | *What our society needs (that RI tools might be able to help with)* | *Compassionate approaches,* |
| Challenges with RI Tools |  | *Issues being faced in being able to implement RI in communities* | *Accessibility, Cultural Differences, Resources* |
|  | Accessibility | *How to make processes more user-friendly and community-serving* | *Technology access, Wifi access, interoperability of systems* |
|  | Communication | *How information is presented and people interpret it* | *Political beliefs, Educational Campaigns, Behavior and Coercion* |
|  | Information | *Accuracy of information and data integrity* | *Timely information, Data Management, Protected Individual Data* |
| Challenges to RI Uptake |  | *Issues in how RI is implemented* | *SVI, Up-to-Date Data* |
|  | Centralization of Resources | *Resources which support RI being readily available* | *Technology, WiFi, community centers* |
|  | Measuring Resilience | *How resilience can be measured to ensure that it is being practiced* | *Community surveys and assessments* |
| Data Sovereignty |  | *Data Management and Data Integrity issues* | *Ensuring privacy of health info and respecting Indigenous data sovereignty* |
|  | Disinformation and Misinformation | *Issues having to do with the spread of false information and how resilience informatics may exacerbate or help with such issues* |  |
| Informatics Tools/ What Informatics tools can do |  | *Examples of informatics tools and its potential* |  |
|  | Area of tool use | *Field in which an RI tool may be used* | *Recreational apps, Mental health, Psychological first aid* |
|  | Potential in RI | *What is not being done currently but could be done* | *AI use could be used to help RI, Can get a lot of information, Use of Apps - to encourage resilience and as a threat in communities against resilience* |
| Community Resources |  | *What resources may exist that may be leveraged or aided by RI* | *Community centers (ex: libraries), Safety nets:* *Adoption services, foster care* |
| People with special considerations for RI tools |  |  | *Refugees- Families, pregnant women and children- Low-income- Tribal communities- Tech illiterate- Chronically ill- Unemployed- Those with special needs- Rural* |
| Major concerns / Threats to resilience |  | *Issues that RI tools need to address that threaten resilience* | *Resource Availability* |
|  | Emotional/Mental Health impact of RI tools | *Resilience issues related to emotion and psychology* | *People not caring, lack of empathy, psychological issues* |
|  | Social/political/infrastructure issues | *Resilience issues related to sociological, politics and infrastructure* | *Data security, Transportation, Food deserts* |
|  | Climate-related issues | *Resilience issues related to the climate* | *Heat, Migration* |
|  | Post-COVID considerations | *Issues that arose due*  *to the COVID-19 pandemic related to resilience* | *Centralization of Resources, Political strain and polarizing terms* |
|  | Interrelated issues | *Issues related to resilience that affect different factors, perhaps in an exacerbating and circular way* | *Transportation, climate change, heat* |
